# Lung adenocarcinoma organoids harboring EGFR 19Del and L643V double mutations respond to osimertinib and gefitinib: A case report

**DOI:** 10.1097/MD.0000000000024793

**Published:** 2021-03-19

**Authors:** Yanan Bie, Jin Wang, Linmin Xiong, Dong Wang, Jing Liao, Yelin Zhang, Hang Lin

**Affiliations:** Wenzhou University, Wenzhou, China.

**Keywords:** epithelial growth factor receptor double mutation, EGFR tyrosine kinase inhibitors, lung cancer organoid

## Abstract

**Introduction:**

It has been well reported that non-small-cell lung cancer (NSCLC) patients with single epithelial growth factor receptor (EGFR) activating mutation have high objective response rate when treated with EGFR-TKIs. However, due to rarity of cases, the response of patients with EGFR double or multiple mutations is not yet well understood. Patient-derived organoid technology has become to a powerful tool in cancer personalized medicine.

**Patient concerns:**

A 60-year-old nonsmoking female was admitted to hospital for lung cancer after Chest CT.

**Diagnoses:**

The patient had no obvious clinical symptoms. Postoperative pathology confirmed a stage I of NSCLC. An EGFR double mutation 19Del/L643V was detected in the sequence of patient's cancer specimen.

**Interventions:**

The patient was in good condition after surgical resection, with no sign of lung cancer recurrence. The patient has not yet started on targeted medicine.

**Outcomes:**

A lung cancer organoid culture was established from the cancer tissue of the patient, which recapitulated the morphological and molecular characteristics of cancer tissue. The drug sensitivity test showed that the cancer organoids that retained original mutations were sensitive to anticancer agents osimertinib and gefitinib, while resistant to erlotinib and icotinib.

**Conclusion:**

The uncommon EGFR double mutation exhibits distinctive sensitivities towards different target drugs of EGFR-TKIs. Our findings provide a better understanding of EGFR-TKIs’ effects on patient-derived cancer organoids harboring uncommon EGFR double mutation(s).

## 1 Introduction

Lung cancer is the most commonly diagnosed cancer and leading cause of cancer death in China. Non-small-cell lung cancer (NSCLC) accounts for more than 85%.^[1]^ Primary NSCLC patients who have double mutations in epithelial growth factor receptor (EGFR) are less common.^[2]^ It has been well acknowledged that NSCLCs containing EGFR kinase domain mutations are especially sensitive to EGFR tyrosine kinase inhibitors (EGFR TKIs).^[3]^ However, it is not clear how NSCLC patients harboring an EGFR double mutation respond to EGFR TKIs.

Organoid technology has been remarkably improved over the last decade. Cancer organoid is generated from cancer tissues derived from patients by three-dimensional culture with a reasonably high success rate.^[4]^ It has been reported that cancer organoids can reflect faithfully the drug response of the corresponding patient.^[5]^ Lung cancer organoid cultures closely recapitulate the morphological and genetic features of clinical samples, therefore, it is useful for predicting patient-specific drug response.^[6,7]^

Here, we present a case of early-stage lung adenocarcinoma harboring an EGFR double mutation. A cancer organoid model was subsequently established from the patient's biopsy specimen to evaluate the drug response to EGFR-TKIs.

## 2 Case report

**Figure 1:**
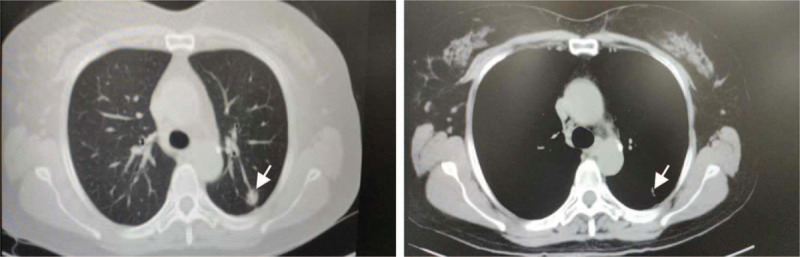
Computed tomography (CT) scan revealed a tumor mass in the upper lobe of left lung (arrow).

A 60-year-old non-smoking female was admitted to hospital for nodules found in her left lung. She had no obvious clinical symptoms. Chest CT (computed tomography scan) scan revealed a mass in the left upper lobe in the left lung (Fig. 1). The patient was admitted for surgery after clinical assessment. In April 2019, the mass (1.7 × 1.1 × 0.9 cm) was removed by thoracoscopic lobectomy. Postoperative pathology confirmed a stage I (pT1N0M0), moderately differentiated adenocarcinoma of NSCLC. Due to the early stage of the lung cancer, the patient has not taken the targeted medicine. The patient was followed up regularly. So far, the patient has been in good condition after surgical resection.

**Figure 2:**
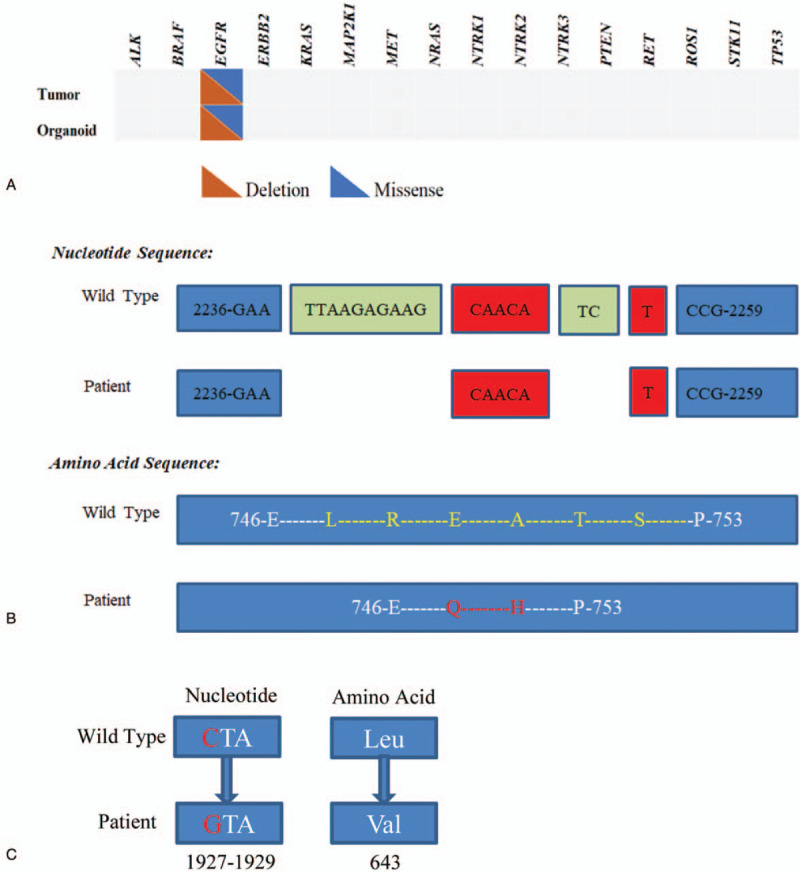
Next-generation sequencing of the organoid and matched tissue. (A). Mutations detected in organoid and matched tissue based on 16 lung cancer genes. (B). Position of in-frame deletion in EGFR exon 19 and comparison with normal EGFR sequence. The deletion sequences/amino acids were shown in green; The insertion sequences/amino acids were shown in red. (C). The schema of nucleotide and amino acid changes of EGFR L643V.

**Figure 3:**
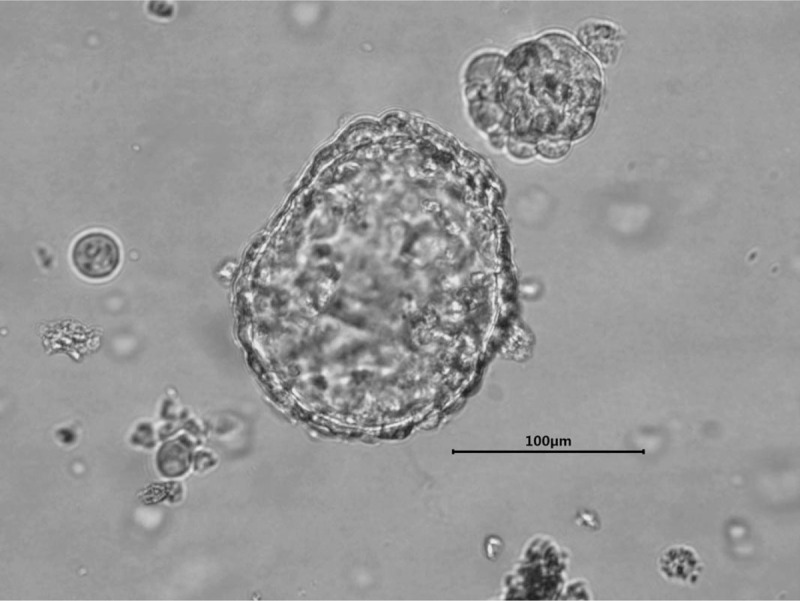
Lung cancer organoid in culture (passage 8).

To select an effective targeted therapy, DNA sequencing study of patient's cancer tissue was carried out and examined using a panel of all exons of 16 genes. An EGFR double mutation 19Del (p.L747_S752delinsQH)/L643V was detected (Fig. 2a). The abundance of the detected EGFR 19Del and L643V mutations were 9.3% and 7.2%, respectively. EGFR exon 19 deletion is a well-known hotspot mutation and highly sensitive to EGFR-TKIs (Fig. 2b). However, L643V, a single point mutation C to G in exon 16 (transcript: Human NM_201284), appeared a new mutation located within transmembrane domain (Fig. 2c). This cancer related mutation is not recorded in most of databases including cosmic, TCGA, ClinVar, EXAC or 1000 Genomes. An organoid model (Fig. 3) was established by culturing the patient's cancer tissue from surgical resection and used for drug screening as described by Sachs.^[6]^ The DNA sequencing result revealed an identical EGFR double mutation 19Del (p.L747_S752delinsQH)/L643V was detected in corresponding cancer organoids (Fig. 2a).

**Figure 4:**
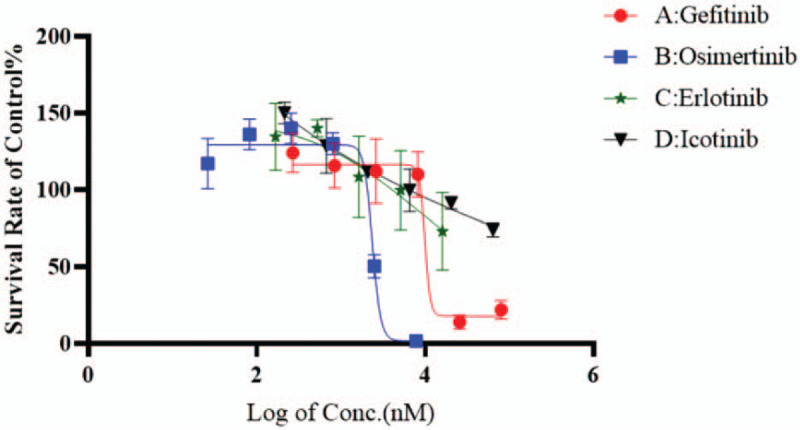
Dose-response curves of the 4 anticancer agents tested in lung cancer organoid culture.

Subsequently, we evaluated the drug response of the lung cancer organoid to EGFR-TKIs. Four first-line drugs for NSCLC treatment, osimertinib, gefitinib, erlotinib, and icotinib, were selected according to the NCCN guideline ^[8]^ and CSCO guideline.^[9]^ The dose-response curve study demonstrated that the EGFR double mutation in cancer organoids was clearly dose responsive to osimertinib and gefitinib with IC50 values of 1.66 μM and 1.26 μM, respectively; but not sensitive to erlotinib or icotinib (Fig. 4).

## 3 Discussion

Targeted therapy is becoming a mainstream in the treatment of advanced NSCLC with oncogenic driver events nowadays. More and more drugs were approved as first-line and subsequent systemic therapy in patients with oncogenic gene mutations.^[10,11]^ And clinicians are now working on developing more targeted therapies. In addition, early-stage NSCLCs are at risk of recurrence even after complete surgical resection. Thus, trials for adjuvant targeted therapy have been conducted to improve survival.^[12]^ It is also important to prospectively predict the treatment response for personalized precision medicine.

In this paper, we presented a case of a female NSCLC patient whose pathological stage was T1N0M0. To learn what gene mutation(s) she possesses and whether she can benefit from targeted therapy, we conducted the DNA sequencing analysis. A double mutation of EGFR (19Del and L643V) was identified in her cancer tissue. So far, the patient has been in her good condition after surgical resection, and yet, no sign of lung cancer recurrence. It suggests that the EGFR double mutation may have a good prognosis.^[13]^ Deletions in exon 19 are the most commonly found EGFR kinase domain mutation in NSCLC, and the predictive effects of this drug-sensitive EGFR mutation is well defined.^[14]^ However, the combination of EGFR 2 mutations may have an effect on its drug response to EGFR TKIs.^[15]^ The incidence rate of double mutation of EGFR has been reported in several studies, however, the clinical significance of EGFR double mutation has not been completely confirmed.^[2]^

Traditional two-dimensional cancer cell lines have long been employed as tumor models to test drug sensitivity. However, many drawbacks hamper the 2D model for clinical use, such as, cell line cultures show their inability in simulating organ-specific functions and are lack of genetic heterogeneity. Cancer organoids are developed from cancer tissues and exert potential implementations in evaluation of drug efficacy.^[5,16]^ Vlachogiannis et al ^[5]^ reported that patient-derived organoids (PDOs) can recapitulate patient responses in the clinic, they found 100% sensitivity and 93% specificity in forecasting response to targeted agents or chemotherapy in patients. The study with lung cancer organoids showed that there was high correlation between drug screening with PDOs and patients’ mutation profiles.^[7]^ Using lung cancer organoid model, we demonstrated that the lung cancer organoids retained the EGFR double mutation found in original cancer tissue, which suggests that lung cancer organoid may be utilized to predict the clinical response of the patient to targeted therapy as well as for clinical drug selection. EGFR 19DEL and L858R are considered to confer a favorable treatment response to first-generation and third-generation TKI therapy. Based on EGFR 19Del, we may suggest that all EGFR-TKIs are effective for the patient. Kim et al ^[7]^ assessed the in vitro patient-specific drug sensitivity with lung cancer organoids (LCO), 2 LCOs had EGFR L858R mutations showed different responses to erlotinib. LCO-43 displayed high sensitivity to erlotinib, while LCO-51 was resistant to erlotinib. This different response to erlotinib could be associated with an intrinsic resistance mechanism, amplification of MET. In our study, EGFR-TKIs sensitivity assessment illustrated that the lung cancer organoids respond to osimertinib and gefitinib notably, while icotinib and erlotinib have partial or little effect on the cancer organoids harboring the uncommon EGFR double mutation. It indicates that the differential response of the lung cancer organoids to different EGFR-TKIs may be associated with the uncommon EGFR double mutation. Further verification are required in future large-scale study.

In summary, the uncommon EGFR double mutation may possess distinctive sensitivities toward different target drugs of EGFR-TKIs. Our findings provide a better understanding of EGFR-TKIs in patients with an EGFR double mutation.

## 4 Conclusion

A patient-derived lung cancer organoid model was established and utilized in anticancer drug screening. The results indicated that the NSCLC organoid line with EGFR double mutations of 19Del and L634V is significantly sensitive to osimertinib and gefitinib.

## Author contributions

**Conceptualization:** Yanan Bie, Hang Lin.

**Data curation:** Yanan Bie, Dong Wang, Jing Liao.

**Investigation:** Yanan Bie, Linmin Xiong, Yelin Zhang.

**Methodology:** Dong Wang.

**Validation:** Jin Wang.

**Writing – original draft:** Yanan Bie.

**Writing – review & editing:** Hang Lin.
